# Mechanism of Fibrotic Anastomosis Formation in Endoscopic Ultrasound‐guided Hepaticogastrostomy Using a Plastic Stent: Insights From an Autopsy Case of Perihilar Cholangiocarcinoma

**DOI:** 10.1002/deo2.70350

**Published:** 2026-05-19

**Authors:** Kosuke Maehara, Junji Mitsushita, Tomoyoshi Shida, Kazuki Hirano, Daisuke Hattori, Yoshiki Sato, Rikako Koyama, Yasuro Miura, Yutaka Takazawa, Tsunao Imamura

**Affiliations:** ^1^ Department of Gastroenterology Toranomon Hospital Tokyo Japan; ^2^ Department of Diagnostic Pathology Toranomon Hospital Tokyo Japan; ^3^ Okinaka Memorial Institute for Medical Research Tokyo Japan

**Keywords:** anastomosis, endoscopic ultrasound‐guided hepaticogastrostomy, endosonographically created route, pathology, perihilar cholangiocarcinoma

## Abstract

Endoscopic ultrasound‐guided hepaticogastrostomy (EUS‐HGS) is an established alternative to other biliary drainage techniques for treating malignant biliary obstruction. Although the anastomosis created by EUS‐HGS is believed to mature within several weeks, histopathological evidence supporting this assumption remains limited, particularly in procedures using plastic stents. Here, we report a case of perihilar cholangiocarcinoma in which an endosonographically created route (ESCR) formed by EUS‐HGS using a plastic stent was histologically evaluated at autopsy. An 83‐year‐old man underwent EUS‐HGS with a 7‐Fr plastic stent for recurrent biliary obstruction after duodenal stenosis, which made endoscopic retrograde cholangiography infeasible. The patient died of disease progression 141 days after the initial EUS‐HGS, and an autopsy was performed. Macroscopic evaluation revealed adhesions between the liver and stomach at the ESCR site. Masson's trichrome staining revealed a well‐defined concentric fibrotic tunnel surrounding the stent tract, with fibrotic thickness comparable to the stent diameter. Fibrotic changes varied across tissue layers, being minimal at the mucosal penetration site and most pronounced at the subserosal and serosal levels. Previous reports of EUS‐guided biliary drainage with self‐expandable metallic stents suggest that radial force contributes to anastomosis maturation. In contrast, the present case demonstrates that a robust fibrotic ESCR can develop even after EUS‐HGS with a plastic stent lacking sustained radial force. These findings provide pathological evidence for structural stabilization of ESCR and support the structural basis for trans‐ESCR procedures.

## 1 Introduction

Recently, endoscopic ultrasound‐guided biliary drainage (EUS‐BD) has been widely adopted as an alternative to other biliary drainage techniques for treating malignant biliary obstruction. EUS‐guided hepaticogastrostomy (EUS‐HGS) is among the most commonly performed procedures. When stent occlusion occurs after EUS‐HGS, stent exchange is required; in this setting, it is crucial that maturation of the endosonographically created route (ESCR) has been achieved. Based on accumulated clinical experience, the anastomosis created by EUS‐HGS is generally believed to mature within approximately 4 weeks [1]; however, histopathological evidence supporting this assumption is limited. In addition, various novel procedures utilizing ESCR have been introduced recently [2]. These trans‐ESCR procedures are expected to impose greater mechanical stress on the anastomosis, making histological maturation and stabilization of the ESCR an essential prerequisite. Histopathological evaluation of the anastomosis after EUS‐HGS in humans has been reported in only one case [3], in which a self‐expandable metallic stent (SEMS) was used. However, no histological evaluations of ESCR after EUS‐HGS using plastic stents have been reported.

Here, we present the autopsy findings of a patient with perihilar cholangiocarcinoma who underwent EUS‐HGS with a plastic stent. We specifically evaluated the pathological characteristics of the ESCR formed during EUS‐HGS.

## 2 Case Report

**FIGURE 1:**
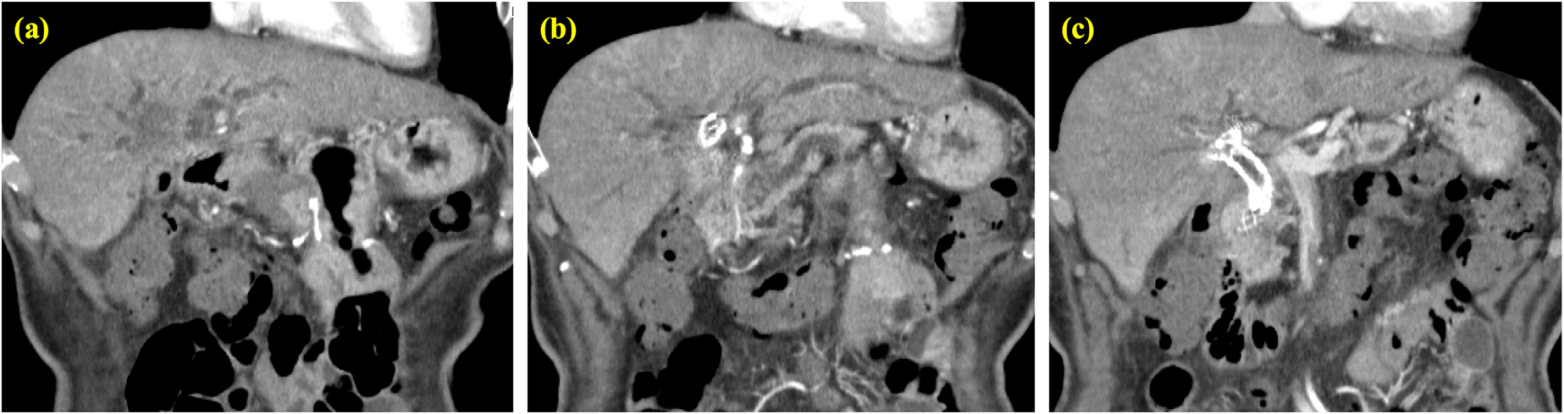
Contrast‐enhanced computed tomography before endoscopic ultrasound‐guided hepaticogastrostomy (EUS‐HGS). (a) A tumor extending from the hepatic hilum to the right hepatic lobe is observed. (b, c) Dilatation of the left intrahepatic bile duct was evident. No ascites was present, and the upper gastric body along the lesser curvature was closely apposed to the liver.

An 83‐year‐old man was referred for further evaluation after an elevated lesion was detected in the bile duct on abdominal ultrasonography. A detailed examination led to a diagnosis of perihilar cholangiocarcinoma (Figure 1). The patient subsequently underwent radiotherapy that year, after which the best supportive care was provided according to his wishes. As the tumor progressed, biliary obstruction and cholangitis developed, and an uncovered SEMS was placed across the hilar bile duct using endoscopic retrograde cholangiography (ERC).

**FIGURE 2:**
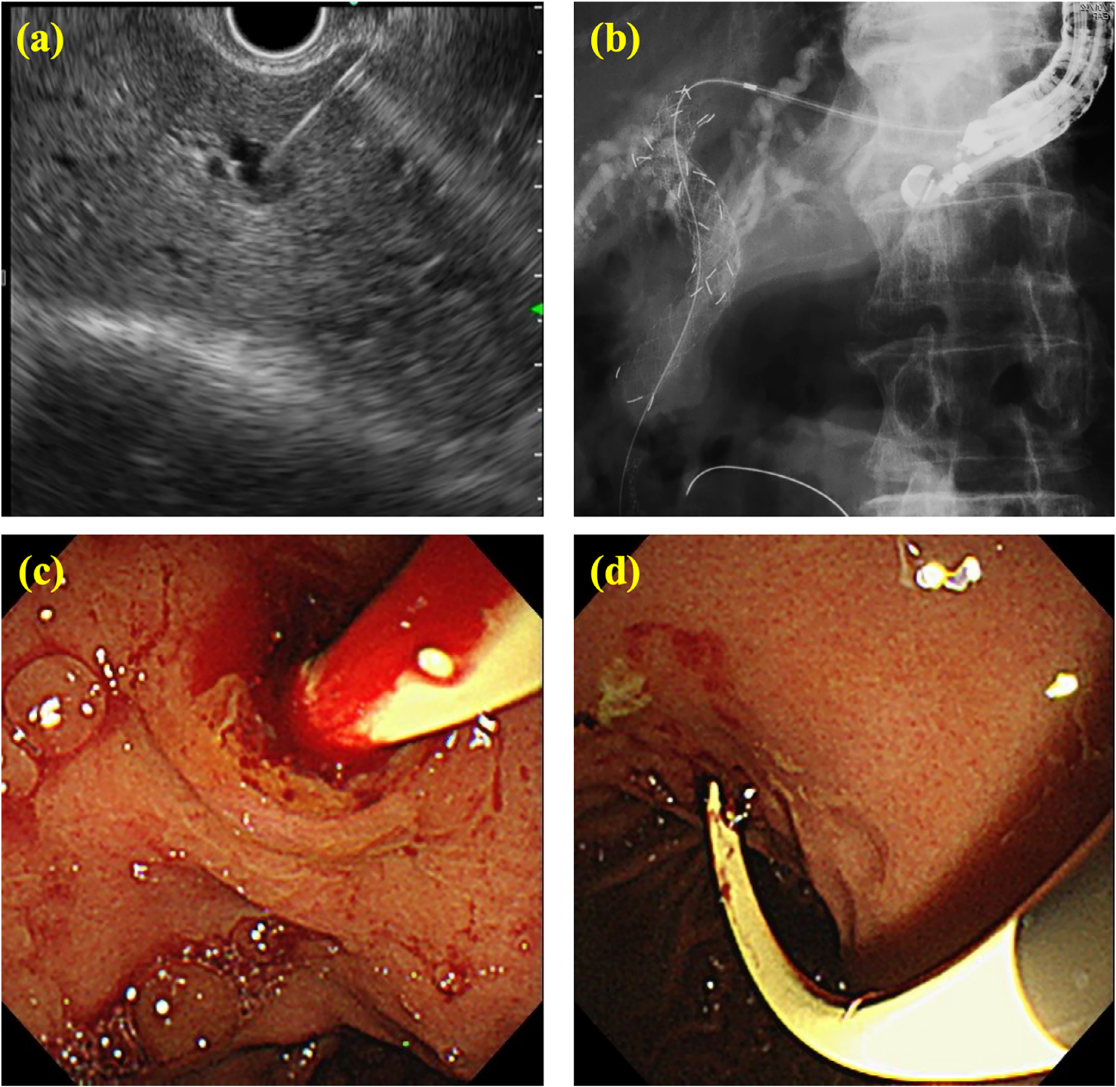
Initial endoscopic ultrasound‐guided hepaticogastrostomy (EUS‐HGS) procedure. After blunt dilation, a 7‐Fr plastic stent was inserted. (a) Puncture of the B3 bile duct from the stomach. (b) Blunt tract dilation. (c, d) Plastic stent deployment in the stomach.

Subsequently, tumor progression caused duodenal stenosis, which rendered ERC technically challenging. Thirty‐one months after the initial diagnosis, EUS‐HGS was performed for recurrent biliary obstruction. Using a 19‐gauge needle (EZ Shot 3 Plus; Olympus, Tokyo, Japan), the B3 bile duct was punctured from the lesser curvature of the upper gastric body. Successful access to the bile duct lumen was achieved with a single puncture. After advancing the guidewire toward the hilar bile duct, the tract was dilated using a 7‐Fr blunt dilator (ES Dilator Soft Type; ZEON Medical, Tokyo, Japan), followed by placement of a 7‐Fr, 14‐cm plastic stent (Through & Pass TYPE IT; Gadelius Medical K.K., Tokyo, Japan) (Figure 2).

Ninety‐three days after the initial EUS‐HGS, stent occlusion was suspected. Using the established ESCR, an uncovered SEMS (8 mm × 4 cm; BileRush Selective; Piolax Medical Devices, Kanagawa, Japan) with a 5.7‐Fr delivery system was placed to bridge the right and left hepatic ducts without additional tract dilation. Subsequently, a 7‐Fr, 14‐cm plastic stent was reinserted through the ESCR, as previously described. No intraprocedural or postprocedural adverse events occurred during these interventions, and no ascites were observed at any time. The patient died of disease progression 141 days after the initial EUS‐HGS.

### 2.1 Macroscopic and Histological Findings at Autopsy

A solid tumor measuring 7.5 × 3.0 × 1.5 cm was identified in the hepatic hilum. The tumor extensively invaded the hepatic parenchyma and showed marked spread along the Glisson's sheath. Extensive peritoneal dissemination accompanied by severe pyloric stenosis was also observed. Peritoneal implants infiltrated the muscularis propria of the gastric antrum. Extensive necrosis was observed throughout the right hepatic lobe, involving both tumor and non‐tumor regions.

**FIGURE 3:**
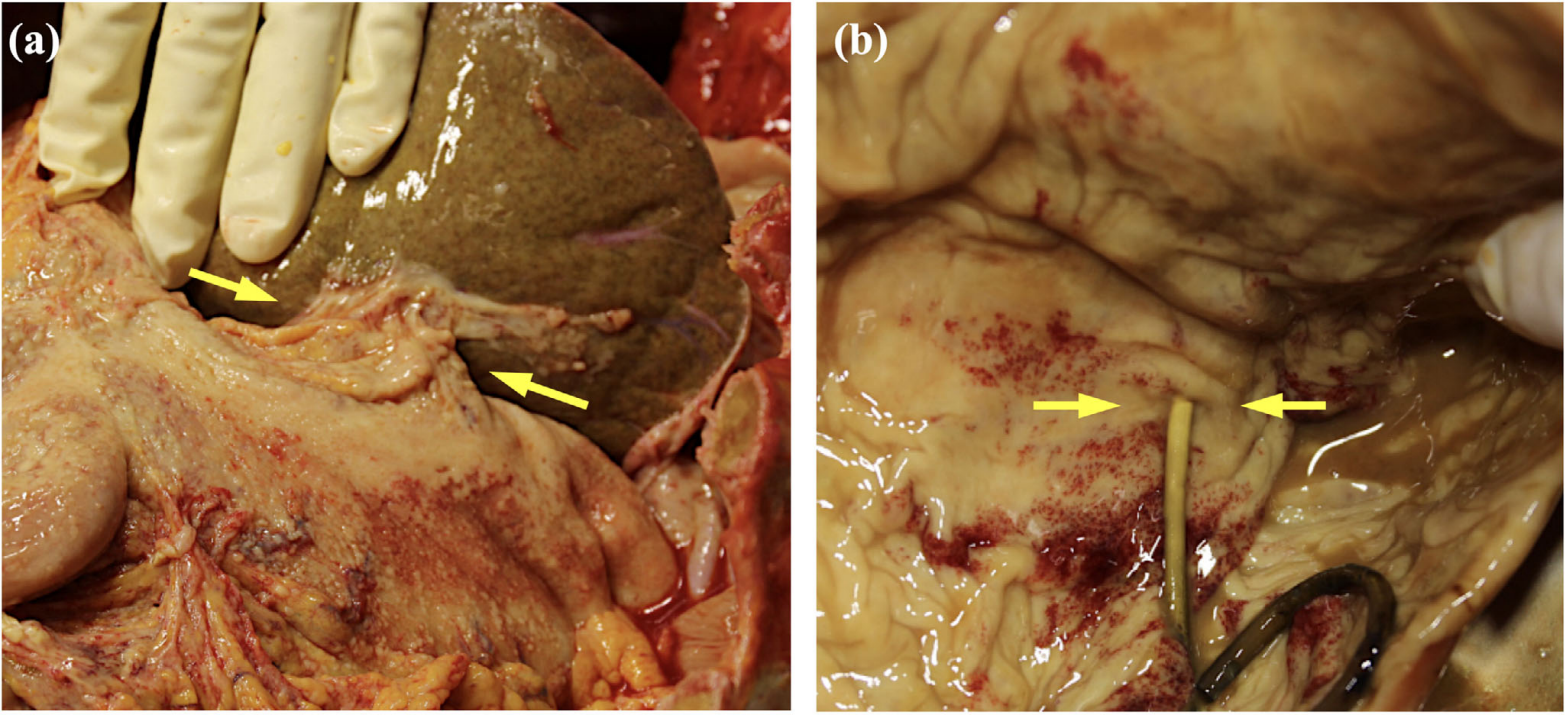
Macroscopic autopsy findings. (a) Hepaticogastric anastomosis: adipose tissue of the lesser omentum adherent to and surrounding the anastomotic site. The arrow indicates the endosonographically created route (ESCR) as viewed from the peritoneal side. (b) Gastric stent entry site: punctate hemorrhages observed in the gastric mucosa, without elevation or induration at the stent entry site. The arrow indicates the ESCR as viewed from the gastric lumen.

Macroscopic examination revealed a cord‐like structure between the liver and stomach, corresponding to the ESCR. The serosal surfaces of the liver and stomach adhered to the tract (Figure 3a). The gastric mucosa at the ESCR entry site showed minimal changes, with no evident induration or elevation (Figure 3b).

**FIGURE 4:**
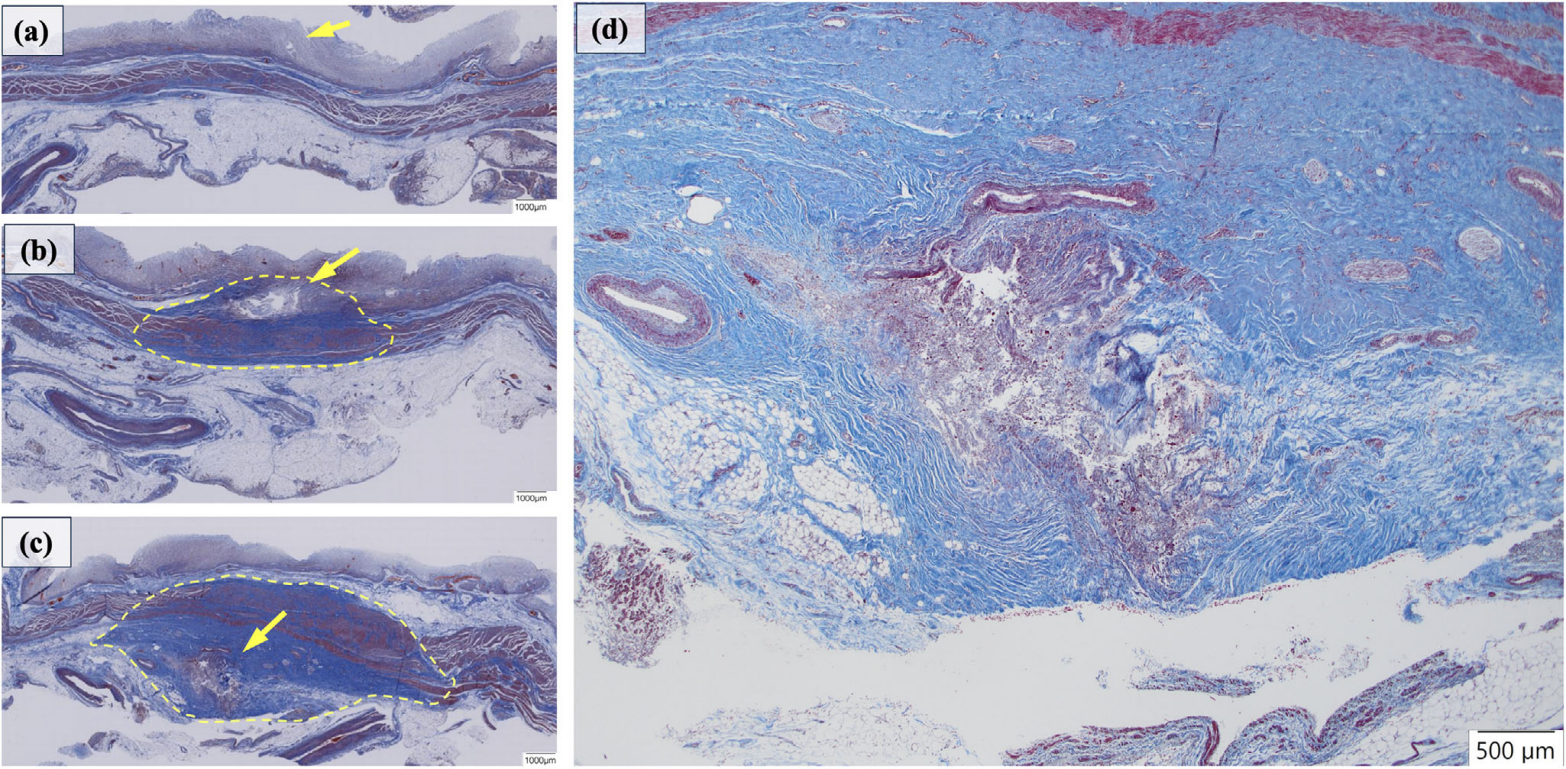
Histological findings at the gastric wall penetration site of the endoscopic ultrasound‐guided hepaticogastrostomy (EUS‐HGS) stent on Masson's trichrome staining. Fibrosis differs by tissue layer. The arrow indicates the center of the tract at the stent penetration site. (a) Mucosal penetration site showing minimal fibrosis. (b) Penetration from the submucosa to the muscularis propria showing moderate peritract fibrosis (yellow dashed lines). (c) Subserosal penetration site showing the most prominent fibrosis (yellow dashed lines). (d) High‐power view of (c).

Histologically, fibrotic changes were not clearly evident on hematoxylin and eosin (H&E) staining (Figure S1); however, the addition of Masson's trichrome staining enabled clear visualization of collagen fibers (Figure 4). On Masson's trichrome staining, minimal fibrosis was observed in the lamina propria and submucosa at the ESCR gastric wall penetration site (Figure 4a). In contrast, marked fibrosis was observed within the muscularis propria at the penetration site (Figure 4b). At the subserosal penetration site, concentric blue‐stained collagen deposition appeared substantially thicker than at other sites, radiating from the stent tract (Figure 4c). Concentric fibrotic tissue with a thickness comparable to the stent diameter (approximately 2300 µm) was observed to form a tunnel‐like structure extending from the serosal surface toward the liver (Figure 4d and Figure S2).

## 3 Discussion

Histopathological assessment of ESCR after EUS‐HGS has been reported in only a single case, in which a SEMS was used. This previous report demonstrated that mature fibrotic tissue on the hepatic and gastric serosal surfaces contributes to anastomosis stabilization after EUS‐HGS using an SEMS [3]. This phenomenon is thought to result in part from the continuous radial force exerted by the SEMS on the surrounding tissues, which induces chronic inflammation and subsequent fibrosis [4]. Consistent with these findings, animal studies evaluating SEMS‐based EUS‐BD have shown remodeling associated with the wound‐healing process, including loss of the gastrointestinal muscular layer and prominent fibroblast proliferation, accompanied by surrounding scar‐like fibrosis [5, 6].

Based on these observations, we hypothesized that ESCR fibrosis would be mild with plastic stents [7], lacking sustained radial force. However, the autopsy revealed thick concentric fibrosis around the stent tract. Fibrosis varied by tissue layer, being minimal at the mucosa and most pronounced at the subserosal and serosal levels, where a robust fibrotic tunnel formed.

Fujita et al. reported histological evaluation of the anastomotic site after EUS‐guided choledochoduodenostomy with a plastic stent [8]. In contrast, our study used the EUS‐HGS route, evaluated a later time point (141 vs. ∼14 days), and included Masson's trichrome staining. While their study focused on early maturation, our findings demonstrate more pronounced and stable fibrosis at 141 days, providing insight into long‐term maintenance for repeated trans‐ESCR procedures.

During wound healing, the acute inflammatory phase is followed by a reparative phase driven by fibroblast proliferation and extracellular matrix deposition, leading to fibrosis [9]; a similar process is thought to occur in the gastric wall in this case. Additionally, the predominance of fibrosis in the serosal and subserosal layers may be explained by minute, clinically undetectable bile leakage early after EUS‐HGS, before complete anastomosis maturation. Such microleakage between the liver and stomach could have induced localized chemical irritation and inflammation, which may have contributed to the strongest inflammatory and fibrotic responses at this site, eventually resulting in the formation of a well‐defined fibrotic tunnel. These findings suggest that in plastic stent–based EUS‐HGS, factors other than stent mechanical properties, such as localized inflammation, may significantly contribute to anastomosis formation.

When evaluating fibrotic maturation of ESCR, the potential impact of subsequent trans‐ESCR procedures should also be considered. In this case, at the time of stent exchange, 93 days after initial EUS‐HGS, no additional dilation was performed, and only a SEMS with a 5.7‐Fr delivery system was placed across the hilar bile duct. Therefore, the influence of this trans‐ESCR procedure on fibrotic tunnel formation was minimal in this patient.

Our histological findings demonstrated that a well‐developed fibrotic tunnel can form between the liver and stomach even after EUS‐HGS with a plastic stent lacking radial force. This provides pathological evidence supporting the structural basis of ESCR and may provide a rationale for both EUS‐HGS and subsequent trans‐ESCR procedures.

This report has several limitations. It remains unclear whether the fibrotic tunnel can withstand the mechanical stress of trans‐ESCR procedures, requiring further investigation. In addition, because this evaluation was performed at autopsy 141 days after EUS‐HGS, early‐phase changes in anastomosis maturation could not be assessed. Further studies are needed to clarify the time course and reproducibility.

## Author Contributions

All authors met all four ICMJE criteria.

**Substantial contributions to the conception or design of the work, or acquisition, analysis, or interpretation of data for the work**: Kosuke Maehara, Junji Mitsushita, Tomoyoshi Shida, Kazuki Hirano, Daisuke Hattori, Yoshiki Sato, Rikako Koyama, Yasuro Miura, Yutaka Takazawa, and Tsunao Imamura.

2) **Drafting the work or revising it critically for important intellectual content**: Kosuke Maehara, Junji Mitsushita, Tomoyoshi Shida, Kazuki Hirano, Daisuke Hattori, Yoshiki Sato, Rikako Koyama, Yasuro Miura, Yutaka Takazawa, and Tsunao Imamura.

3) **Final approval of the version to be published**: Kosuke Maehara, Junji Mitsushita, Tomoyoshi Shida, Kazuki Hirano, Daisuke Hattori, Yoshiki Sato, Rikako Koyama, Yasuro Miura, Yutaka Takazawa, and Tsunao Imamura.

4) **Accountable for all aspects of the work, ensuring that accuracy or integrity matters related to any part of the work are appropriately investigated and resolved**: Kosuke Maehara, Junji Mitsushita, Tomoyoshi Shida, Kazuki Hirano, Daisuke Hattori, Yoshiki Sato, Rikako Koyama, Yasuro Miura, Yutaka Takazawa, and Tsunao Imamura.

## Funding

The authors have nothing to report.

## Conflicts of Interest

The authors declare no conflicts of interest.

## Ethics Statement

Written informed consent for autopsy and publication of anonymized clinical information and images was obtained from the patient's family in accordance with institutional and national ethical standards.

## Supporting information




**FIGURE S1** Histological findings at the gastric wall penetration site of the EUS‐HGS stent on hematoxylin and eosin (H&E) staining. The arrow indicates the center of the tract at the stent penetration site. (a) Mucosal penetration site. (b) Penetration from the submucosa to the muscularis propria. (c) Subserosal penetration site.

**FIGURE S2** Layer‐dependent differences in fibrosis at the stent penetration site. The arrow indicates the center of the tract. (a) Mucosal penetration site showing minimal fibrosis. (b) Subserosal penetration site showing marked fibrosis.
