# Supplementary figures and images for: Mechanism of Fibrotic Anastomosis Formation in Endoscopic Ultrasound‐guided Hepaticogastrostomy Using a Plastic Stent: Insights From an Autopsy Case of Perihilar Cholangiocarcinoma
Source: DEN Open. 2026 May 19;7:e70350. doi: 10.1002/deo2.70350 (PMC13184719; doi:10.1002/deo2.70350)

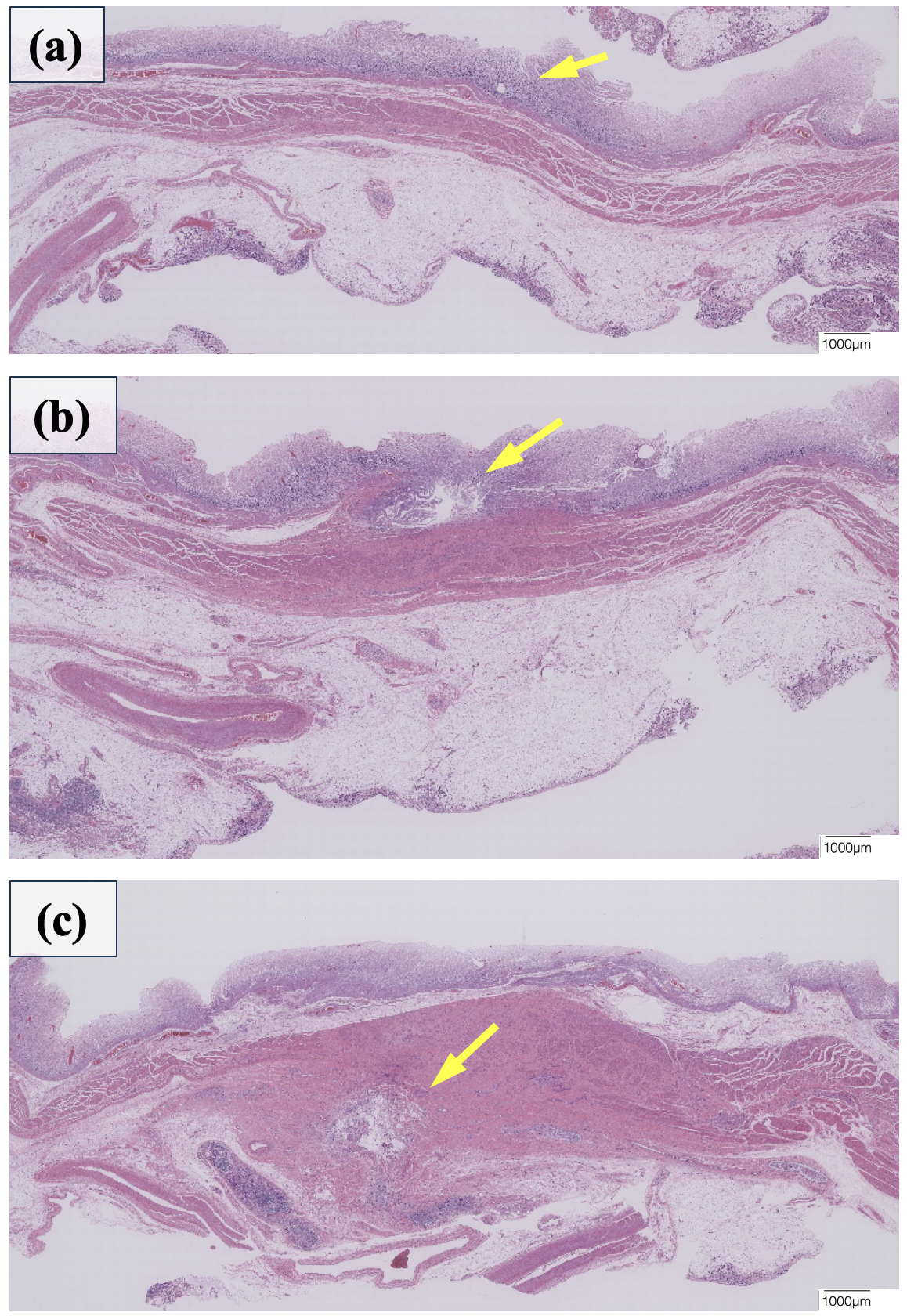

Supplement: Supplementary file 1 — FIGURE S1 Histological findings at the gastric wall penetration site of the EUS‐HGS stent on hematoxylin and eosin (H&E) staining. The arrow indicates the center of the tract at the stent penetration site. (a) Mucosal penetration site. (b) Penetration from the submucosa to the muscularis propria. (c) Subserosal penetration site. [file DEO2-7-e70350-s001.png]

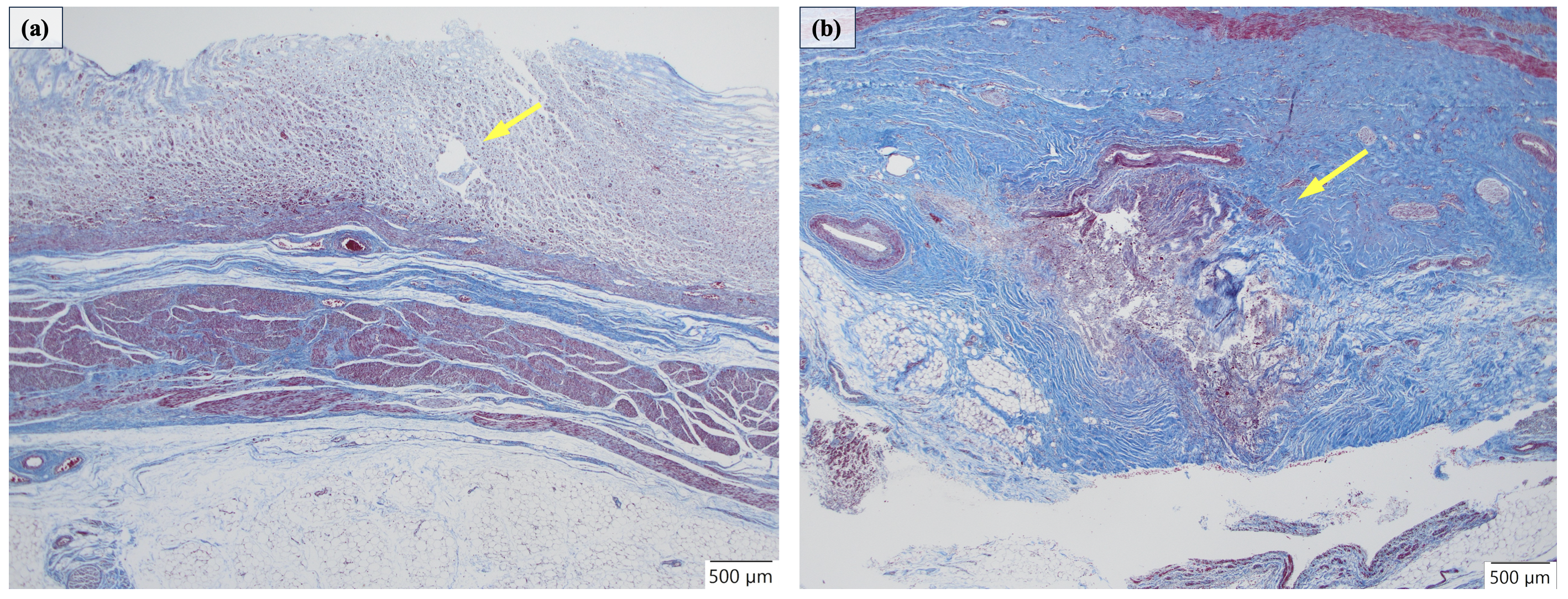

Supplement: Supplementary file 2 — FIGURE S2 Layer‐dependent differences in fibrosis at the stent penetration site. The arrow indicates the center of the tract. (a) Mucosal penetration site showing minimal fibrosis. (b) Subserosal penetration site showing marked fibrosis. [file DEO2-7-e70350-s002.png]
